# Supplementary material for: Hopf Bifurcation in Mean Field Explains Critical Avalanches in Excitation-Inhibition Balanced Neuronal Networks: A Mechanism for Multiscale Variability
Source: Front Syst Neurosci. 2020 Nov 26;14:580011. doi: 10.3389/fnsys.2020.580011 (PMC7725680; doi:10.3389/fnsys.2020.580011)
Supplement: Supplementary file 1 [file Presentation_1.pdf]

Supplementary Material of

Hopf Bifurcation in Mean Field Explains Critical Avalanches in Excitation-Inhibition Balanced Neuronal Networks: A Mechanism for Multiscale Variability

Junhao Liang , Tianshou Zhou and Changsong Zhou

Appendix 1. Model Extensions

1.1 Extension to time-varying inputs.

The field model Equations (11), (12) can be conveniently used to compute the transient firing rate dynamics of the network in response to the time-varying external input. For inhomogeneous Poisson external inputs with time-dependent firing rate $Q_o(t)$, the constant term Q_o in Equation (12) can just be modified to the time-dependent term $Q_o(t)$ and the field model can be simulated directly in this way. Note that a good estimation of the effective parameter σ_E, σ_I may depend on Q_o, as the estimation result given by Equation (10) depends on Q_o. However, we point out that if $Q_o(t)$ does not change very strongly, the parameters σ_E, σ_I can be kept constant throughout the change of $Q_o(t)$ once they have been estimated. To demonstrate this, we set a time-varying input

$$Q_o(t) = \begin{cases} \lambda \ , t \in [0, 200) \\ 4\lambda \ , t \in [200, 400) \\ 2\lambda \ , t \in [400, 500) \\ 2\lambda(1 + \sin[\frac{\pi}{100}(t - 500)]) \ , t \in [500, 800] \end{cases} \tag{S1}$$

with $\lambda = 5Hz$ for each neuron as shown in **Supplementary Figure S1A**. Here, we do not consider the synaptic transmission delay and the synaptic decay times are set as $\tau_d^E = \tau_d^I = 4\ ms$, $\tau_r = 0\ ms$, where the network would be in asynchronous dynamics. Note that the external input $Q_o(t)$ contains constant part, discontinuous jumps and continuous changes. Simulations show that the firing rate changes accordingly respondent to the change of input, as can be seen from the raster plot in **Supplementary Figure S1B**. At the same time, we can simulate the field model Equations (11), (12) with the same time-varying input $Q_o(t)$ and with fixed parameters σ_E, σ_I used in **Figure 2B**. As shown in **Supplementary Figure S1C**, the field model predicts the changing trend of the average firing rate of the network. It should be noted that this simple scheme ignores some complex nature of the firing rate response properties in the presence of synaptic filtering (Moreno-Bote and Parga, 2004; Ledoux and Brunel, 2011).

1.2 Extension to conductance-based models.

The present mean-field theory can be directly generalized to conductance-based (COB) model where the postsynaptic inputs received by each neuron depend on the membrane potential of the neuron. Specifically, we further study a COB model with the dynamic equation Equation (1) replaced by

$$\frac{dV_i}{dt} = f_\alpha(V_i) + (V_E^{rev} - V_i)[g_{\alpha o} \sum_{j\in\partial_i^o} F^E * s_j(t-\tau_l^E) + g_{\alpha E} \sum_{j\in\partial_i^E} F^E * s_j(t-\tau_l^E)] + (V_I^{rev} - V_i) g_{\alpha I} \sum_{j\in\partial_i^I} F^I * s_j(t-\tau_l^I) \quad \text{(S2)}$$

Here, the reversal potential for excitatory and inhibitory synaptic currents are $V_E^{rev} = 0\ mV$ and $V_I^{rev} = -70\ mV$ respectively. The synaptic strengths of conductance are set as $g_{EO} = 0.025$, $g_{IO} = 0.04$, $g_{EE} = 0.02$, $g_{IE} = 0.04$, $g_{EI} = 0.27$, $g_{II} = 0.48$. Other notations, parameters and settings are the same as the current-based (CUB) case. Similar to the CUB model, the COB model shows emergence of collective oscillation induced by slow inhibition. Such a critical transition can also be predicted by our mean-field theory as a Hopf bifurcation, while the derivation of the field equation is slightly different. In the CUB case, the field equation Equation (12) can be obtained by taking the average $\langle . \rangle_\alpha$ of the original equation Equation (1) under mean-field assumption. In the COB case, we still can take the average $\langle . \rangle_\alpha$ of the Equation (S2) under mean-field assumption, but have to proceed with the decoupling assumption that

$$\langle V_i [g_{\alpha E} \sum_{j\in\partial_i^E} F^E * s_j(t-\tau_l^E) + g_{\alpha I} \sum_{j\in\partial_i^I} F^I * s_j(t-\tau_l^I)] \rangle_\alpha \approx \langle V_i \rangle_\alpha \langle g_{\alpha E} \sum_{j\in\partial_i^E} F^E * s_j(t-\tau_l^E) + g_{\alpha I} \sum_{j\in\partial_i^I} F^I * s_j(t-\tau_l^I) \rangle_\alpha \quad \text{(S3)}$$

This is based on the fact that in an E-I balanced network where neurons spike irregularly, one expects that at any given time t, the correlation between the membrane potential and the recurrent E, I conductance input for different neurons is small. As such, we get the field equations

$$\frac{dV_\alpha}{dt} = f_\alpha(V_\alpha) + (V_E^{rev} - V_\alpha)\left[g_{\alpha o}\left(n_o Q_o + \sqrt{\frac{n_o Q_o}{N_\alpha}}\xi_\alpha(t)\right) + g_{\alpha E}\Phi_E\right] + (V_I^{rev} - V_\alpha) g_{\alpha I}\Phi_I \ , \alpha = E, I \quad \text{(S4)}$$

to replace Equation (12), where $\Phi_\alpha(t) = \langle \sum_{j\in\partial_i^\alpha} F^\alpha * s_j(t-\tau_l^\alpha) \rangle_{E,I}$ still obeys Equation (11). Thus, Equations (11), (S4) constitute the field equations of the COB model Equation (S2). The sigmoid relation Equation (9) can still be assumed and σ_E, σ_I can be estimated in a numerical way through Equation (10). A summarization and comparison between the field equations of CUB model Equation (1) and COB model Equation (S2) is as follows.

CUB:

$$\begin{cases} \frac{dV_\alpha}{dt} = f_\alpha(V_\alpha) + J_{\alpha o}\left(n_o Q_o + \sqrt{\frac{n_o Q_o}{N_\alpha}}\xi_\alpha(t)\right) + J_{\alpha E}\Phi_E + J_{\alpha I}\Phi_I \\ \left(\tau_d^\alpha \frac{d}{dt} + 1\right)\left(\tau_r \frac{d}{dt} + 1\right)\Phi_\alpha = \frac{n_\alpha}{\left[1+\exp\left(\frac{V_{th} - V_\alpha(t-\tau_l^\alpha)}{\sigma_\alpha}\frac{\pi}{\sqrt{3}}\right)\right]}, \ \alpha = E, I \end{cases} \quad \text{(S5)}$$

COB:

$$\begin{cases} \frac{dV_\alpha}{dt} = f_\alpha(V_\alpha) + (V_E^{rev} - V_\alpha)\left[g_{\alpha o}\left(n_o Q_o + \sqrt{\frac{n_o Q_o}{N_\alpha}}\xi_\alpha(t)\right) + g_{\alpha E}\Phi_E\right] + (V_I^{rev} - V_\alpha) g_{\alpha I}\Phi_I \\ \left(\tau_d^\alpha \frac{d}{dt} + 1\right)\left(\tau_r \frac{d}{dt} + 1\right)\Phi_\alpha = \frac{n_\alpha}{\left[1+\exp\left(\frac{V_{th} - V_\alpha(t-\tau_l^\alpha)}{\sigma_\alpha}\frac{\pi}{\sqrt{3}}\right)\right]}, \ \alpha = E, I \end{cases} \quad \text{(S6)}$$

The calculation of the steady-state and its stability analysis at zero transmission delays can be performed in the same way as in CUB model. The qualitative results are similar to the CUB model. There is a critical value τ_d^{I*} such that when $\tau_d^I < \tau_d^{I*}$ the steady-state is a stable focus, corresponding to the asynchronous strict balance state of the network. For $\tau_d^I > \tau_d^{I*}$, the steady-state destabilizes through a supercritical Hopf bifurcation (**Supplementary Figure S1D**), corresponding to the onset of collective oscillation in the network, as shown by the PCC in **Supplementary Figure S1E**. The spiking of individual neurons are still irregular, as can be seen from the high CV of ISIs in **Supplementary Figure S1F**. Furthermore, near the Hopf bifurcation point, the COB model exhibits critical properties in terms of avalanche dynamics similar to the results of CUB model. Overall, the quality of theoretical prediction in the COB case is worse than the CUB case. Indeed, the COB input would lead membrane potential more bias to a Gaussian distribution (Richardson and Gerstner, 2005), an assumption in our derivation. A complete analytical treatment of COB model is very challenging (Renart et al., 2004). However, the semi-analytical mean-field approach here constitutes an effective description of the macroscopic dynamics of E-I network, which has an advantage that it works for both CUB and COB dynamics.

Supplementary Reference

Ledoux, E., and Brunel, N. (2011). Dynamics of networks of excitatory and inhibitory neurons in response to time-dependent inputs. *Front. Comput. Neurosci.* 5, 25.

Moreno-Bote, R., and Parga, N. (2004). Role of synaptic filtering on the firing response of simple model neurons. *Phys. Rev. Lett.* 92, 28102.

Renart, A., Brunel, N., and Wang, X.-J. (2004). Mean-field theory of irregularly spiking neuronal populations and working memory in recurrent cortical networks. *Comput. Neurosci. A Compr. approach*, 431–490.

Richardson, M. J. E., and Gerstner, W. (2005). Synaptic shot noise and conductance fluctuations affect the membrane voltage with equal significance. *Neural Comput.* 17, 923–947.

Appendix 2. Sensitivity of the Critical Points on the Effective Parameters

The mean-field scheme to derive the field equations introduces two effective parameters σ_E, σ_I to construct the voltage-dependent firing rate relation Equation (9) and they are the crucial quantities that determine the quality of the scheme. Thus, it is important to know how the theoretically predicted critical point τ_d^{I*} depends on the choice of σ_E, σ_I.

Although the critical point in the field model can be thought as a Hopf bifurcation point, the concept of critical point is not decisive in the E-I spiking neuronal network. This can be seen from **Figure 2D** and **Supplementary Figure S1B** which show that the spiking correlation increases in a somewhat continuous way as τ_d^I increases. As a rough approximation, we define the critical point in the E-I spiking network as the parameter value where the distance of the avalanche size distribution to its best fit power law distribution, D defined in Materials and Methods, is minimal, as shown in **Figure 3C**. We stress that the critical properties of avalanche shown in **Figure 3** are statistical properties so that for parameters close enough to this critical value, critical properties can still maintain in a statistically significant manner. However, if there are several avalanche data sets with sufficient evidence to claim criticality, to judge which one is closer to criticality is still a challenging open issue. We find that for large network size, if σ_E, σ_I are estimated in the numerical way through Equation (10), the critical point in the spiking network, is very close to the Hopf bifurcation point in the field model. We denote τ_d^{I*} as the Hopf bifurcation point under this 'optimal' estimation of the parameters σ_E, σ_I using Equation (10).

We compute the Hopf bifurcation point ${\tau_d^I}^{Hopf}(\sigma_E, \sigma_I)$ predicted by the field model for different values of σ_E, σ_I and compare it to the 'real' critical point (estimated by τ_d^{I*}). The difference ${\tau_d^I}^{Hopf}(\sigma_E, \sigma_I) - \tau_d^{I*}$ of the CUB model and the COB model can be seen in **Supplementary Figure S2**. From **Supplementary Figure S2**, we can see that once σ_E, σ_I are estimated with suitable values, such as by Equation (10), the prediction of the critical synchronous transition point is very precise.

We also notice that the bifurcation point predict by the field model seems to mainly depend on the difference $\sigma_E - \sigma_I$. Once $\sigma_E - \sigma_I$ lies on suitable ranges, the predicted critical point will be very close to the 'real' one. It can also be noticed that in the CUB model the critical point is not sensitive to the values of σ_E, σ_I compared with the COB model, as can be seen from **Supplementary Figure S2A to C** that the difference ${\tau_d^I}^{Hopf}(\sigma_E, \sigma_I) - \tau_d^{I*}$ is still low for a large range of parameter values. On the contrary, the sensitivity in the COB case implies that the COB model has more complicated intrinsic dynamic nature that has to be further explored.

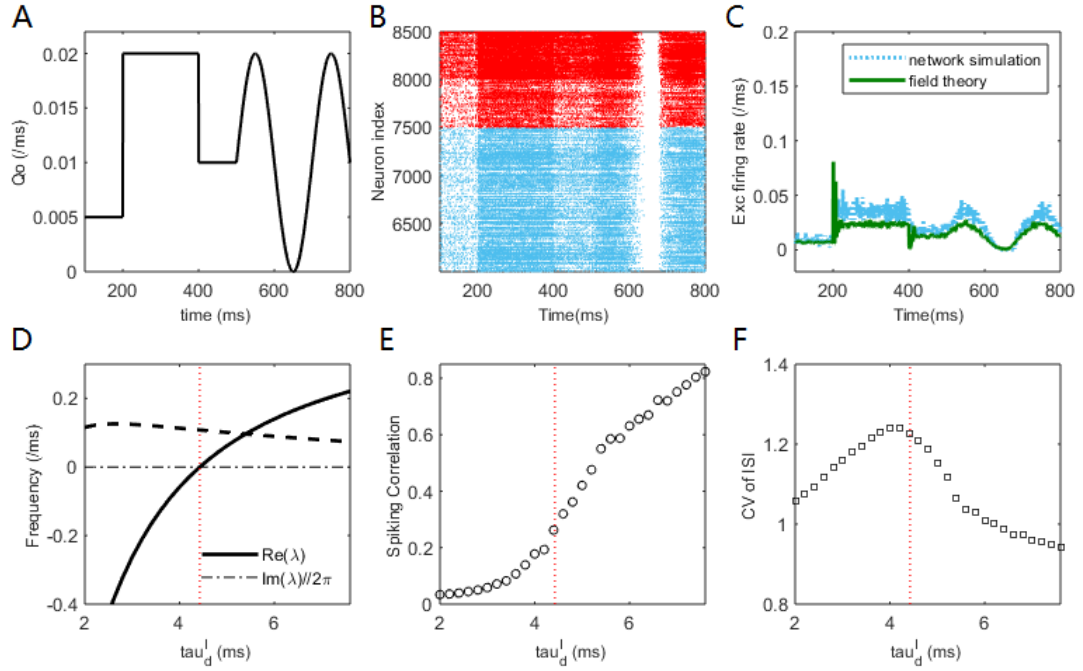

Supplementary Figure 1. Results of the generalized models. (A-C) CUB network dynamics in response to time-varying external input. **(A)** The time-dependent input function $Q_o(t)$ used in simulation. **(B)** Raster plot of the spiking time of neurons (only part of the N =10000 neurons are shown). The excitatory/ inhibitory neurons are indicated in blue/red. **(C)** Comparison of the mean firing rate of excitatory population obtained by network simulation and field model simulation. **(D-F)** Mean-field theory prediction of the transition from asynchronous to synchronous state in COB model. **(D)** Field equations predict that a Hopf bifurcation occurs as the increase of inhibitory decay time τ_d^I at a critical value around $\tau_d^I \approx 4.4\ ms$. The real and imaginary part (divided by 2π) of the dominant eigenvalue are given by the solid and dashed lines, respectively. **(E)** The PCC index shows the emergence of network oscillation as the increase of τ_d^I across the bifurcation point. **(F)** The CV of ISI at different value of τ_d^I. The parameters in COB model are set as $\tau_l^E = \tau_l^I = 0\ ms$, $\tau_d^E = 2\ ms$, $\tau_r = 0\ ms$ and $Q_o = 5\ Hz$.

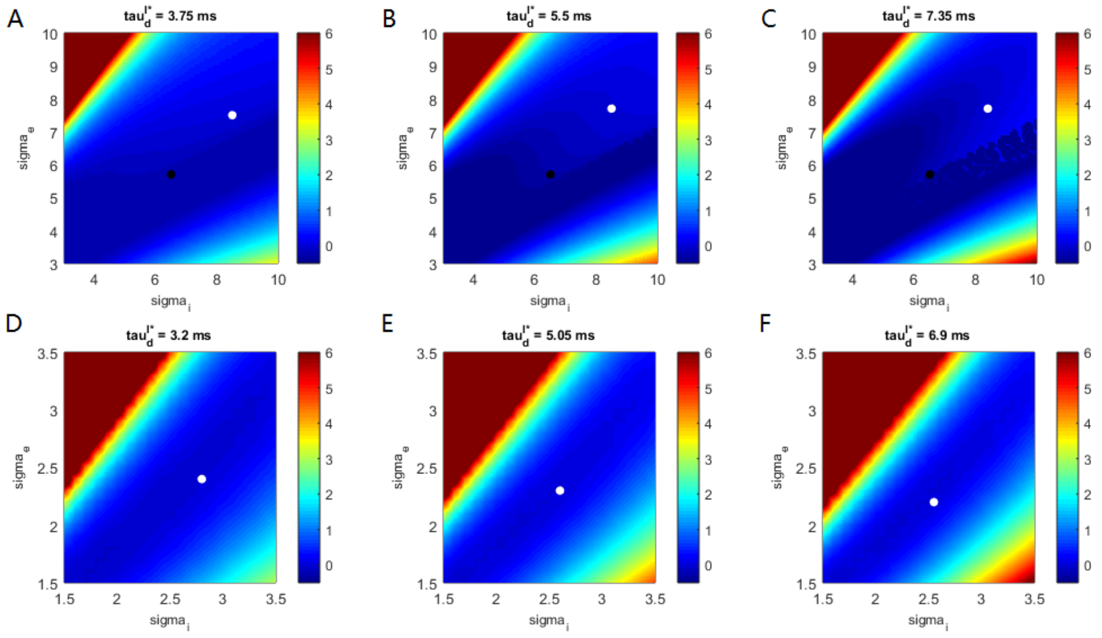

Supplementary Figure 2. Sensitivity of the predicted critical point on the effective parameters. The difference value $\tau_d^{I\ Hopf}(\sigma_E, \sigma_I) - \tau_d^{I*}$ is shown by color for current-based (CUB) model **(A-C)** and conductance-based (COB) model **(D-F)**. White dots in **(A-F)** indicate the positions of the numerical estimation results by Equation (10) and the corresponding estimated critical value τ_d^{I*} is shown on top of the plots. Black dots in **(A-C)** indicate the positions of the theoretical estimation results by fixed σ_E, σ_I used in **Figure 2B** in the CUB model. Parameters are $\tau_d^E = 2\ ms$ for **(A, D)**, $\tau_d^E = 3\ ms$ for (**B, E**), $\tau_d^E = 4\ ms$ for (**C, F**) and $Q_o = 5\ Hz$ for all the cases.

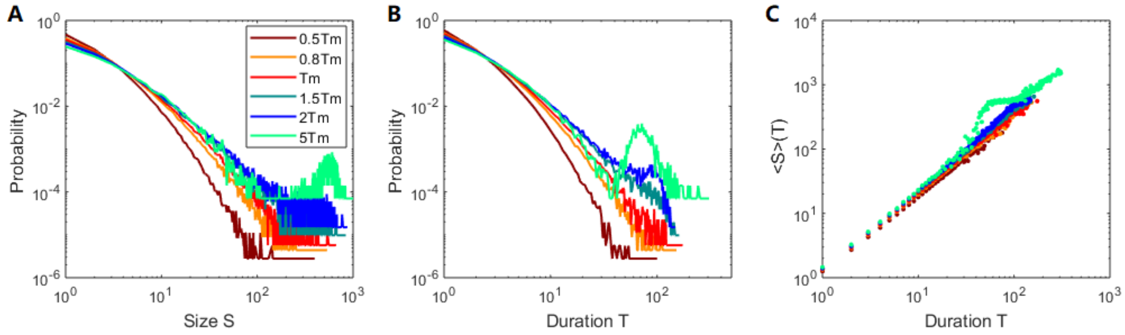

Supplementary Figure 3. Effect of using different time bins for measuring avalanches. We measure the avalanches of CUB model at critical point $\tau_d^I = 3\ ms$ with $Q_o = 5\ Hz$ with different sizes of time bin. **(A)** The probability density distribution of the avalanche size. **(B)** The probability density distribution of the avalanche duration. **(C)** The mean avalanche size with respect to a given avalanche duration. Different colored curves are avalanche distributions constructed with time bins labeled in **(A)**. We have used the average ISI of the merged spiking train, T_m, as the time bin in **Figure 3** in the main text. Here, we further compare the results from using $0.5T_m$ to $5T_m$. It can be seen that the usage of T_m produces very good critical properties and the results are similar when using time bins that are not deviated too much from T_m. However, using a too large time bin will induce a 'bump' at large value in the avalanche distribution since this captures a large oscillating scale of the network.

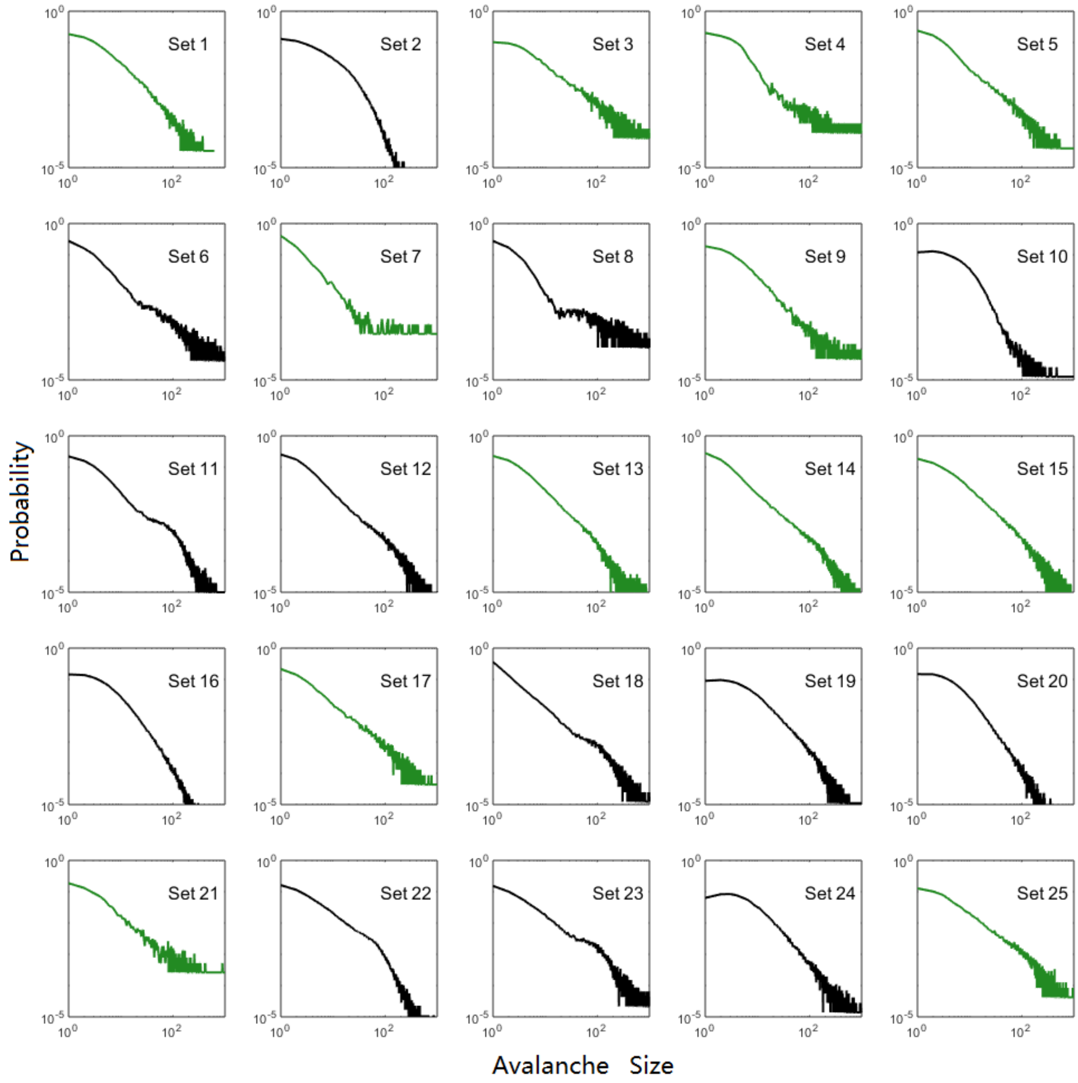

Supplementary Figure 4. Avalanche size distributions of the data sets. According to our standard, the up-state of data Set 1,3,4,5,7,9,13,14,15,17,21,25, plotted by green color, exhibit significant power-law size distribution $P(S) \sim S^{-\tau}$. Refer to **Supplementary Table 1** for Details.

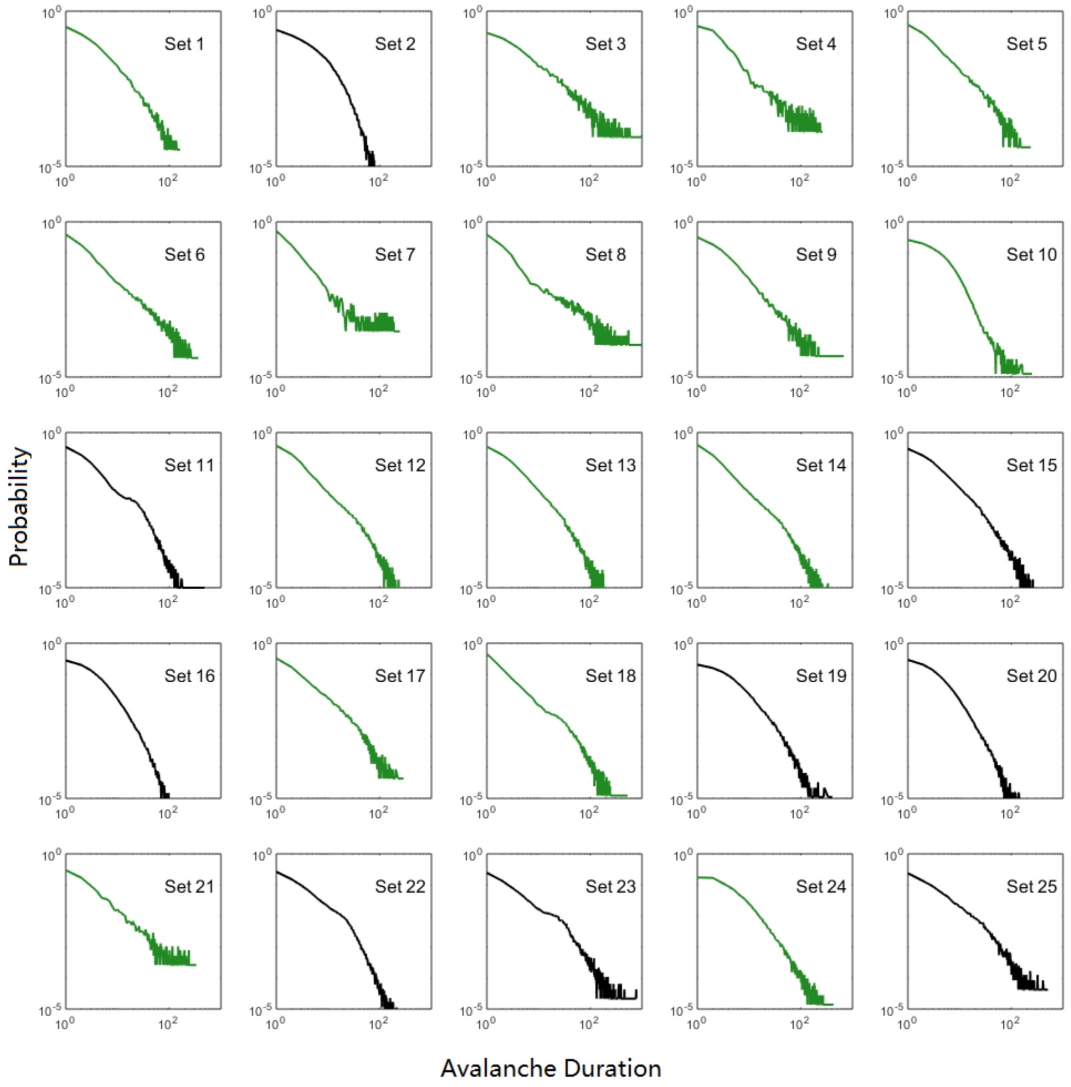

Supplementary Figure 5. Avalanche duration distributions of the data sets. According to our standard, the up-state of data Set 1,3,4,5,6,7,8,9,10,12,13,14,17,18,21,24, plotted by green color, exhibit significant power-law duration distribution $P(T)\sim T^{-\alpha}$. Refer to **Supplementary Table 1** for Details.

Supplementary Table 1. Estimating the critical exponents of the data sets. The number of neurons, length of the up-states, the time bin used in measuring the avalanches, maximum avalanche size and duration, those estimated critical exponents, data ranges after truncations and the p-values in KS test of the fitted power laws in each data set are shown. Here, the avalanche size and duration values are in their original linear scale.

Set No.	Neuron amounts	Up-state length (mins)	bin size (ms)	max size	max duratio	size range	τ	p value	duration range	α	p value	1/σνz	(α-1)/(τ-1)
1	166	12.60	3.70	627	162	[10,95]	1.78	0.118	[9,50]	2.183	0.108	1.218	1.226
2	443	28.24	2.05	476	115								
3	99	9.16	1.97	11502	1970	[4,125]	1.248	0.42	[4,52]	1.365	0.406	1.245	1.472
4	98	8.08	5.62	1713	264	[12,144]	1.223	0.18	[11,71]	1.349	0.516	1.223	1.565
5	367	11.35	3.53	1090	237	[7,124]	1.419	0.188	[4,25]	1.644	0.846	1.321	1.537
6	286	10.06	2.20	1798	365				[7,52]	1.442	0.88	1.271	
7	172	4.90	6.43	2230	245	[2,27]	1.837	0.984	[2,14]	2.088	0.804	1.327	1.300
8	191	8.06	2.19	8002	1304				[6,116]	1	0.644	1.204	
9	333	7.51	2.37	2775	677	[5,105]	1.751	0.212	[5,44]	1.95	0.878	1.309	1.265
10	368	17.56	2.44	1222	251				[9,59]	3.431	0.114	1.366	
11	243	16.79	1.25	2195	481								
12	346	14.45	1.03	1534	349				[7,50]	1.653	0.252	1.266	
13	381	16.40	1.34	1409	321	[7,116]	1.662	0.334	[4,33]	1.85	0.164	1.284	1.284
14	304	16.24	0.79	2071	450	[7,91]	1.452	0.226	[6,46]	1.599	0.22	1.248	1.325
15	206	16.49	1.07	2066	445	[7,90]	1.566	0.704					
16	594	22.72	0.87	1230	278								
17	435	10.07	2.75	1566	287	[7,113]	1.309	0.356	[3,24]	1.424	0.794	1.372	1.372
18	180	14.46	1.08	3280	525				[2,17]	1.621	0.146	1.310	
19	262	11.58	0.88	1685	407								
20	534	18.02	1.49	1318	273								
21	444	2.91	3.02	2985	334	[8,117]	1.377	0.806	[2,53]	1.5	0.978	1.326	1.326
22	384	20.85	0.76	2598	487								
23	310	13.79	1.14	5015	779								
24	358	12.33	1.08	2093	429				[13,100]	2.306	0.168	1.235	
25	231	10.71	1.93	3256	510	[5,150]	1.229	0.632					
